# P196: Post-neurosurgical meningitis

**DOI:** 10.1186/2047-2994-2-S1-P196

**Published:** 2013-06-20

**Authors:** S Madan, R Soman, N Gupta

**Affiliations:** 1 Medicine and Infectious Diseases, PD Hinduja National Hospital and Medical Research Centre, Mumbai, India

## Introduction

Post-neurosurgical meningitis represents only 0.4% of all nosocomial infections. However, it presents enormous diagnostic and therapeutic challenge with increasing rates of multidrug resistant organisms. Hence strict infection control is of paramount importance.

## Objectives

To study the etiology, management and risk factors of post-neurosurgical meningitis.

## Methods

A prospective and retrospective observational study of six patients admitted over a period of one year, who developed meningitis following various neurosurgical procedures. Antimicrobial treatment was initiated empirically based on clinical and epidemiological background and later modified based on results of culture and susceptibility. Retrospectively, risk factors associated with each case were analyzed.

## Results

Patients had undergone either intracranial or spinal neurosurgical procedures with placement of ventricular or spinal drains. Features of meningitis developed between 3 days to 2 months after surgery. Various organisms isolated were *Staphylococcus aureus*, vancomycin resistant enterococci (VRE), *Escherichia coli*, *Pseudomonas aeruginosa*, *Pseudomonas stutzeri* and *Klebsiella pneumoniae.* Antibiotics used for treatment included cloxacillin, linezolid, daptomycin, meropenem, ceftriaxone, ciprofloxacin and colistin; based on organism identification and susceptibility.

1 patient with lumbar drain related meningitis, treated with meropenem and intrathecal colistin showed dramatic response. 1 patient with external ventricular drain (EVD) related meningitis caused by *Klebsiella pneumoniae* and another with infected pseudomeningocele by *Pseudomonas stutzeri*, also improved. 3 patients did not respond well and expired. Of these, 1 had infection with VRE, and 2 had incomplete source control with retention of foreign implants.

Risk factors presumed to have role in etiology and poor outcomes were uncontrolled diabetes, obesity, non-removal of ventriculoperitoneal shunt, suboptimal duration of antimicrobial treatment, disconnection of lumbar drain; prolonged duration (>5 days), blockage and multiple changes of EVD. Factors related to surgical technique like intraoperative dural tear and injury to lumbar vertebrae were also involved.

## Conclusion

Although post-neurosurgical meningitis is a low incidence infection, the consequences are grave. Therefore, effective prevention with stringent infection control measures should be the goal.

## Disclosure of interest

None declared

